# Promoting Fruit and Vegetable Intake in Parents: A Cluster Randomised Controlled Trial

**DOI:** 10.3390/ijerph18105206

**Published:** 2021-05-13

**Authors:** Yuying Sun, Samantha S. W. Fung, Patrick K. W. Man, Alice N. T. Wan, Sunita Stewart, Tai Hing Lam, Sai Yin Ho

**Affiliations:** 1 School of Public Health, The University of Hong Kong, Hong Kong, China; gyysun@hku.hk (Y.S.); sam.fung@cpce-polyu.edu.hk (S.S.W.F.); director@aka.org.hk (A.N.T.W.); syho@hku.hk (S.Y.H.); 2 School of Professional Education and Executive Development, The Hong Kong Polytechnic University, Hong Kong, China; 3 Tung Wah Group of Hospitals, Hong Kong, China; patrick.man@tungwah.org.hk; 4 Aberdeen Kai-Fong Welfare Association Social Service Centre, Hong Kong, China; 5 Department of Psychiatry, The University of Texas Southwestern Medical Center, Dallas, TX 75390, USA; Sunita.Stewart@utsouthwestern.edu

**Keywords:** primary prevention, randomised controlled trial, behaviour change, dietary intakes, fruit and vegetable

## Abstract

We conducted a cluster randomised controlled trial of parents in 56 primary schools and community service centres (clusters) to evaluate the effectiveness of a single-session workshop on promoting more fruit and vegetable (FV) intake. A total of 803 parents were randomised to the FV intervention arm (16 clusters, *n* = 197), the more appreciation control arm (19 clusters, *n* = 270), or the less criticism control arm (21 clusters, *n* = 336). The FV intake of the FV arm was compared with that of the combined more appreciation or less criticism (MALC) arm. Both arms received a 2 h workshop: (i) the FV arm on increasing FV consumption and related food literacy; (ii) the MALC arm on increasing appreciation or reducing criticism of children. Primary outcomes were FV consumption per day in the past week assessed at baseline, 2-weeks, and 6-weeks. Secondary outcomes were behavioural determinants proposed by the Health Action Process Approach (HAPA), including outcome expectancies, self-efficacy, intention, and planning behaviour. The FV arm had a greater increase in FV consumption than the MALC arm, with large effect sizes (*d*: 0.97–1.08) and improvements in behavioural determinants with small effect sizes at all time points (*d*: 0.19–0.43). Our study was the first population-based randomised controlled trial to show that a brief, single 2 h HAPA-based workshop was effective in promoting fruit and vegetable intake in parents.

## 1. Introduction

Ample evidence shows that fruit and vegetable (FV) intake reduce the risk of chronic health problems including cancers, stroke, cardiovascular diseases, diabetes, obesity, and mortality [1,2,3,4,5,6]. However, the minimum intake of five daily servings of FV recommended by the World Health Organization is not met in most parts of the world [7]. Low consumption of FV contributes to 1.8% of the total global burden of disease and 2.6 million deaths worldwide [8].

A national survey in England found robust evidence of lower mortality for higher FV intake of up to seven servings daily [3]. More recent systematic reviews found lower risk of cardiovascular disease for FV intake of up to 10 servings per day [9,10]. Hong Kong’s Department of Health, in line with the World Health Organization’s guideline, recommends at least five servings of FV per day. However, the Population Health Survey 2014/2015 found that 94.4% of Hong Kong’s people consumed less [11], as did over 89% of adults in the Behavioural Risk Factor Surveys between 2004 and 2016 [12]. The downward fruit intake trends observed among both females and males across all ages are a public health concern [12].

Many factors can affect FV consumption behaviours [13]. Nutritional knowledge may play a small but pivotal role in adopting healthier food habits [14,15]. A local survey found that even though over 70% of respondents were aware of the five-per-day recommendation (“2 Plus 3 Every Day” Fruit and Vegetable Promotional Campaign), 50% to 80% had little knowledge of the actual size of a serving of a fruit or vegetable [16]. Better knowledge may help with a more accurate assessment of one’s own intake and hence may lead to better planning of ways to increase FV consumption.

Food literacy is an increasingly used term to describe the nutritional knowledge and skills required in food planning and management, selection, preparation, and eating [17]. Planning is the key component proposed by the Health Action Process Approach (HAPA) that bridges the intention–behaviour gap and promotes behavioural changes [18]. A few programmes have assessed the effectiveness of brief HAPA-based interventions for increasing FV consumption, but the interventions were intensive [19,20]. Some studies focused on specific populations, such as students [21,22]. To our knowledge, no population-based studies have been conducted. Given the typical busy urban lifestyle in Hong Kong, population-based approaches with brief interventions are more practical and can reach more people because they require fewer resources [23].

We conducted the More Appreciation and Less Criticism (MALC) Project, which was a cluster randomised controlled trial (RCT) with three arms: more appreciation (MA), less criticism (LC), more FV intake. Our innovative RCT design with three arms allowed us to test three types of interventions and outcomes cost effectively. Results comparing the MA and LC arms with the FV arm on parenting outcomes have been published separately [24]. In the present paper, the FV intake of the FV arm was compared with that of the combined MALC arm. It was hypothesized that greater improvements in FV intake and HAPA determinants would be observed in the FV arm than the MALC arm.

## 2. Materials and Methods

This study was a parallel cluster RCT with two arms: FV arm and MALC arm. The study was conducted in partnership with the Tung Wah Group of Hospitals from April 2012 to May 2013. The trial was registered under the Clinical Trials Centre of The University of Hong Kong (HKUCTR-1598, date of registration: 2 April 2013) and ClinicalTrials.gov (NCT04445818, date of registration: 24 June 2020). The detailed process and methods have been published [24]. All methods were carried out in accordance with CONSORT guidelines.

### 2.1. Participants

We invited 168 units (primary schools, parent–teacher associations, and integrated community service centres) in six conveniently sampled districts out of all 18 districts in Hong Kong via letters and telephone calls. Participants were parents of children attending Grade 3–6 of primary schools (aged 8–12). Parents were excluded if they had active psychiatric problems, suicidal thoughts, personality disorders, emotion problems and intellectual disabilities. All participants provided written informed consent. The Institutional Review Board of the University of Hong Kong/Hospital Authority Hong Kong West Cluster (No. UW 12-483) approved the study. We adopted a computer-based randomisation procedure and concealed the random numbers from the cluster representatives and researchers before the enrolment of participants.

### 2.2. Intervention and Control Groups

*Fruit and Vegetable (FV)*. Participants in the FV intervention arm attended a 2 h single-session programme and completed a homework booklet. To enhance food literacy, the intervention was designed for empowerment of the participants to choose more types of fresh FV through learning nutritional knowledge and improving their self-efficacy and planning behaviour. The intervention included information about the minimum amount of daily FV required to form a healthy diet, serving size of common FV (e.g., one serving of fruit equals a medium-sized apple, one serving of a vegetable equals half a rice bowl of a cooked vegetable), and self-efficacy enhancement in consuming at least five servings of FV (e.g., recalling past successful experiences in preparing enough FV) per day. The instructors were social workers or project workers who had received a one-day training workshop before delivering interventions to the participants. In order to ensure that the intervention programmes followed the protocol, standardised training materials were developed and provided to the instructors. Members of the project team were trained to do fidelity checks on the intervention sessions. In general, no deviations were reported for any of the intervention sessions, although the percentage of adherence was not documented.

Participants first watched a short video clip about how to consume a minimum of five servings of FV daily. This was followed by small group discussion involving attributional questions (e.g., think about the long-term effects of eating more fruits and vegetables for themselves and their families). These questions were used to elicit positive outcomes of eating more FV (e.g., better health and bowel movements) or negative outcomes of not eating FV (e.g., higher risks of non-communicable diseases). Participants were encouraged to come up with healthy recipes with FV ingredients and to think of their own plan, writing down where, what, how, and when they could eat more FV.

Participants received a 10-page homework booklet with five parts: (i) importance of consuming FV; (ii) graphs and pictures of a balanced diet and recommended minimum daily intake of FV; (iii) exercises to help participants record the various types of consumed FV in the past seven days; (iv) useful websites for additional reference; and (v) positive changes in diet after joining the project. They were asked to propose an ideal level of FV intake and record the actual intake for two weeks.

*More Appreciation or Less Criticism (MALC)*. Participants in the MALC control arm attended a single-session programme without any content on health or diet (about two hours). The parents watched a 6 min engaging video about expressing appreciation or criticism to children and discussed the positive outcomes of expressing appreciation or the negative effects of criticism. Participants were then asked to plan when, what, and how they would express appreciation or use alternatives to criticism. The details have been reported previously [24].

Participants received a 6-page homework booklet with five parts and were asked to think of or record: (i) expressions of appreciation/impacts of criticism; (ii) the negative consequences of criticizing; (iii) what was worth appreciating in their children/alternatives to criticism—positive coaching; (iv) two hypothetical scenarios to show their appreciation/to practise positive coaching; (v) successful experiences of appreciation/substituting criticism with positive coaching.

### 2.3. Primary Outcome

The primary outcome was self-reported FV intake assessed at baseline and at 2-week and 6-week follow-ups. FV intake was assessed by summing two items: “On average, how many servings of fruit did you eat every day in the past week? One serving is equal to a medium-sized apple, orange, or banana”, and “On average, how many servings of vegetables did you eat every day in the past week? One serving is equal to half a rice bowl of a cooked vegetable, e.g., Chinese flowering cabbage, kale, spinach, cabbage, bean sprouts, eggplant, or carrot”. The Cronbach’s α was 0.76. Cappuccio and colleagues evaluated such a two-item questionnaire to estimate FV intake [25], which is suitable for widespread use in the general population. It has a slightly lower validity than the 24 h dietary recalls but has significant associations with serum carotenoids (*r* = 0.34 vs. 0.42) [26]. The questions were used in the Hong Kong Behavioural Risk Factor Surveys [12].

### 2.4. Secondary Outcomes

Potential mediators of FV intake based on the HAPA model, including outcome expectancies, intention, self-efficacy, action planning (a clear plan on pursuing behavioural goals relevant to the intervention), and coping planning (overcome anticipated barriers to action), were measured with simple or single items. Similar items have been used in previous HAPA-based behaviour change programmes in the West and in Hong Kong [18,27]. Participants rated their agreement on a 10-point scale (1 = totally disagree, 10 = totally agree). We have shown good test-retest reliability of the single-item scale with a score of 0–10 [28].

*Outcome expectancies* was assessed by averaging two items (range 1–10, assessed at baseline and immediate post-intervention): “If I eat more fruits and vegetables every day, I will be healthier” and “If I eat more fruits and vegetables every day, I will set a good example for my children”. *Intention* and *Self-efficacy* were respectively measured by one item (range 1–10, assessed at baseline, immediate post-intervention, and after 2 weeks): “In the coming 2 weeks, I intend to eat more fruits and vegetables”, and “In the coming 2 weeks, I am confident that I can eat more fruits and vegetables”. *Action planning* and c*oping planning* were measured respectively by one item (range 1–10, assessed at baseline, immediate post-intervention, and after 2 weeks and 6 weeks): “In the coming 2 weeks, I have a clear plan on how to eat more fruits and vegetables”, and “In the coming 2 weeks, I have a clear plan on how to overcome challenges to eat more fruits and vegetables”.

### 2.5. Open-Ended Questions

Participants were asked to complete two open-ended questions to evaluate the workshop. The first question asked what the participants liked most about the workshop and the reasons; the second question asked what areas of the workshop required improvement and the reasons.

### 2.6. Statistical Analyses

As the results based on the MA and LC control arms separately were similar (Supplementary Tables S1 and S2), they were combined as one control arm in the present paper to increase the precision of effect estimates. Baseline characteristics of the FV and MALC arms were compared using independent *t*-test and chi-square test where appropriate. 

For the primary analysis, an intention-to-treat approach was used with multiple imputation to replace missing values (mi ice command in Stata 13.1) [29]. A multivariate mixed model was built to calculate between-group mean differences (BMD) in FV intake and HAPA mediators of the two arms at 2-weeks and 6-weeks post-intervention, after adjusting for baseline values of respective outcomes, unbalanced demographics, and clustering effect. Fruit intake and vegetable intake were also reported separately to explore their respective changes. Cohen’s *d* was calculated with values of 0.2, 0.5, and 0.8, indicating small, medium, and large effect size (ES), respectively [30].

We built three models as secondary analyses, which were all post hoc analyses unspecified in the protocol. The first model was built by including an extra interaction term of arm and FV intake at baseline (“4 servings or less” or “5 servings or more” per day) to control for the bias that might be caused by different FV intakes at baseline. The other confounding factors were the same as in the primary analysis. The second model was built by including all the confounding factors and the HAPA variables (outcome expectancies, intention, self-efficacy, action planning, and coping planning) measured at immediate post-intervention and after 2 weeks (if available) to explore the effect of the arm and HAPA variables on the outcomes. The third model was per-protocol sensitivity analyses, including the same confounding factors in the primary analysis but only involving participants who had completed all assessments.

Open-ended questions were analysed using thematic content analysis. All the answers were read thoroughly and coded by a research assistant. Then the data were collated into potential themes. The themes were checked and refined until clear definitions and names were created for each theme. Some compelling examples were extracted.

## 3. Results

### 3.1. Study Sample

**Figure 1:**
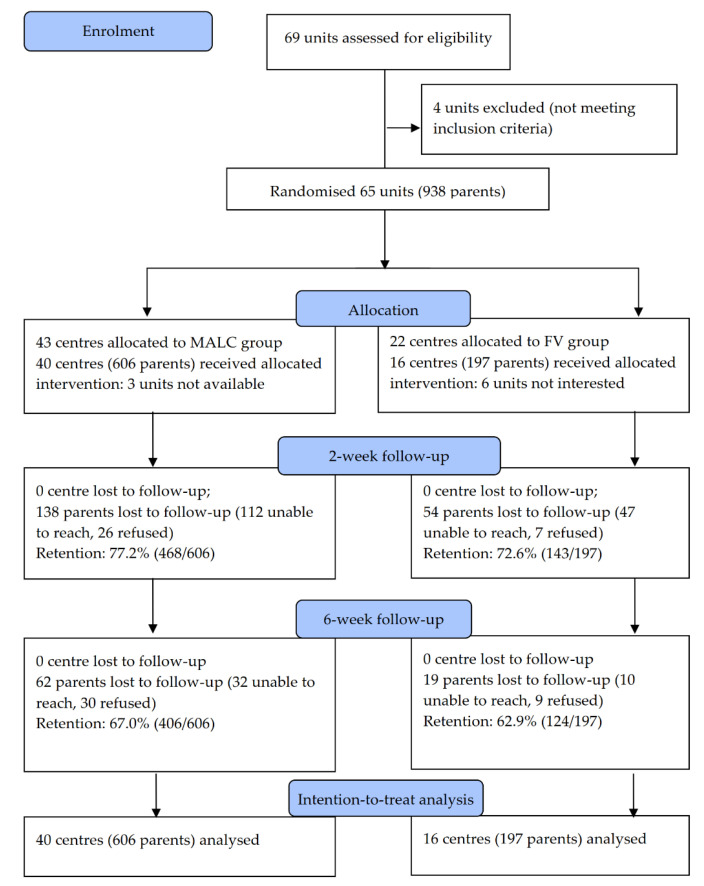
CONSORT flowchart.

A total of 803 participants from 56 randomised clusters provided consent and completed baseline and post-intervention questionnaires (FV: 16 clusters, *n* = 197, MALC: 40 clusters, *n* = 606). Figure 1 shows the CONSORT flow diagram. Table 1 shows participants were mostly female (90.3%) and married (91.0%). Participants in the FV arm had lower education level (chi-square = 7.44, *p* = 0.02), lower household monthly income (chi-square = 16.1, *p* < 0.001), and more children (*t* = 4.41, *p* < 0.001) than those in the MALC arm.

### 3.2. Primary Outcome

The average FV intake per day in the past week was 4.41 servings (SD = 2.71, range 0–16) at baseline (FV: 5.41 servings; MALC: 4.09 servings). The proportion of participants who had consumed less than five servings of FV per day in the past week was 67.0% at baseline (45.2% in FV arm; 74.0% in MALC arm).

Table 2 shows greater increases in FV intake in the FV arm than the MALC arm, with large effect sizes at 2-week and 6-week follow-ups (2-week BMD = 1.78, 95% CI: 1.44 to 2.13, ES = 0.97; 6-week BMD = 1.92, 1.43 to 2.40, ES = 1.08). While increased FV intake was observed within the FV group at 2-week and 6-week follow-ups, FV intake decreased within the MALC arm at both follow-ups. The model including the interaction term also showed significantly more improvement in FV intake in the FV arm than in the MALC arm, but the interaction term had no significant impact on the FV intake. The separate outcomes of fruit intake and vegetable intake in the FV arm both showed significant improvement compared with the MALC arm.

### 3.3. Secondary Outcomes

Table 2 shows greater increases in all the HAPA outcomes in the FV arm than in the MALC arm (Cohen’s *d*: 0.19–0.43). For within-group changes of the FV arm, improvements in the positive outcome expectancies and intention were observed immediately after the intervention. Self-efficacy, action planning, and coping planning in the FV arm showed improvements at all follow-ups. The MALC arm showed improvements in positive outcome expectancies, intention, self-efficacy, action planning, and coping planning immediately after intervention. However, the MALC arm showed decreases in intention, self-efficacy, action planning, and coping planning at 2-week and 6-week follow-ups. For the model including all the HAPA variables, only the arm (FV vs. MALC), FV intake at baseline, educational level, and income had significant impact on the outcomes.

### 3.4. Per-Protocol Analysis

The per-protocol analysis also showed a large effect size of the intervention on the primary outcome. Supplementary Table S3 shows the results of the HAPA variables in the per-protocol analysis (Cohen’s *d*: 0.30–0.46; *n* = 514), which were similar to those of the intention-to-treat analysis.

### 3.5. Open-Ended Questions

After the workshop, 111 participants in the FV arm answered the open-ended questions. Regarding the favourite part of the workshop, 31 participants chose writing recipes with FV because they enjoyed creativity with meal planning, being able to share thoughts and to learn from other participants, and learning new ways of cooking. “*The part on designing recipes helps unleash our creativity.*” Learning about the amount of one serving of a fruit or vegetable was regarded as the favourite activity by 19 participants. “*Calculating the servings of fruit and vegetables helps participants monitor how much they have eaten.*” Thirteen participants liked the introduction to nutrition facts the most. “*Introducing nutrition combination can improve participants’ family eating habits.*” 

Three participants suggested that there could be more examples of recipes, whereas another three participants indicated that the workshop did not recommend strategies that induced and maintained changes in eating habits. “*How can one maintain consuming five servings of fruit or vegetables every day?*”

Regarding the perceived effect, all participants (*n* = 109) said that the workshop was helpful at 6-week follow-up, 25.7% reported that they had consumed more FV after attending the workshop (“*The whole family has increased intake of fruit and vegetables.*”), 22.9% thought that the workshop served as a reminder to eat more FV *(*“*It can remind me to eat more fruit and vegetables and consume at least five servings a day.*”*).*

## 4. Discussion

The present study shows that the HAPA-driven single-session intervention was effective in promoting self-reported FV intake and HAPA-related behavioural determinants in a sample of Hong Kong Chinese parents with small to large effect sizes (*d*: 0.19–1.08). According to the qualitative feedback, the participants were satisfied with and enjoyed the programme.

The population-based approach, also called the universal approach, addresses the entire population rather than high risk groups. The universal approach may yield a smaller effect size compared with the targeted or treatment approach [31]. Some of our previous studies in Hong Kong supported this viewpoint [27,32]. Our previous paper showed small to moderate effect sizes of the single-session interventions for increasing parental appreciation and decreasing criticism [24]. Therefore, it is encouraging to achieve large effect sizes of a single-session FV intervention in improving the FV intake behaviours (0.97 after 2 weeks and 1.08 after 6 weeks). This was achieved despite a higher level of baseline FV intake in the FV arm (5.41 servings per day) than the control arm (4.09 servings per day), as making further increase in the FV arm would have been more difficult. Because we adopted cluster RCT, the individuals were aware of the intervention group assignments when we enrolled them. Cluster randomisation might not be as good as individual randomisation in achieving balanced baseline measurements and demographics. The average FV intake in the intervention arm reached the recommended FV level at baseline, but our results showed that there was still room for improvement. The homework assigned to the intervention group might have had a positive effect on the FV intake as it clearly showed graphs and pictures of FV in one serving size, acting as a reminder for the participants. Because up to 10 servings of FV per day are still beneficial for preventing cardiovascular diseases [9,10], the interventions for improving FV consumption could still be appropriate for those who had consumed five servings. The significantly decreased FV intake within the MALC group after 2 weeks and 6 weeks might have contributed to the large between-group effect size. Yet the reason for such a decrease is unclear and could be the result of natural changes during the follow-up period. Furthermore, some non-specific factors might have been involved, such as interaction and communication with the instructor. The magnitude of these factors might not have been balanced and were hard to estimate. We assumed these factors could have caused limited impact on the FV outcomes although they could have improved family wellbeing [24].

Previous studies showed that behavioural change could be affected by the HAPA determinants [33]. We observed positive changes in the FV arm in HAPA-related behavioural determinants at all time points with small effect sizes (*d*: 0.19–0.43) compared with the MALC arm. Since the behavioural change had a more substantial change than the HAPA variables, the mechanism underlying behavioural change seemed to be largely direct, and cognitive changes played only a minor role.

A survey reported 50% to 80% of Hong Kong residents had knowledge of the actual serving size of FV. Our trial indicated the importance of this knowledge. Although there have been campaigns to improve the awareness of the five-per-day recommendation, people may still be unclear about the actual serving size. Therefore, this should be emphasised in future health promotion programmes that attempt to reach more people and increase FV intake. Given the considerable variation in the time taken to reach the limit of automaticity and the formation of a habit (18 to 254 days) [34], booster sessions are needed to achieve a sustainable effect and a higher proportion of adequate daily servings at the population level. A one-session workshop conducted by social workers (not dietitians) receiving a one-day training workshop can be low-cost and easily delivered, benefitting more parents in activities held in social service centres or schools. Although social workers might not be experienced in delivering nutrition knowledge to clients, they could gain a broader understanding of nutrition and confidence through such a simple training programme. They may include the knowledge into their future healthy eating programmes and activities without the presence of dietitians. Additionally, with knowledge about serving sizes and frequency, the other components of food literacy could be added to future programmes, such as skills needed in food selection and preparation [17].

This study had several limitations. Firstly, participants were aware of the intervention they received and self-reported data were used (as in most behavioural intervention trials). Such awareness might have led to social desirability bias with differential over-reporting of FV intake in follow-up assessments. On the other hand, we could not sort out the potential random error and bias of the observed effect size. However, because our qualitative data did show that some subjects reported improvements, the effect size was unlikely to be wholly due to bias. The significantly decreased FV intake in the MALC group in follow-ups indicates that social desirability bias was unlikely. Secondly, because long questionnaires might decrease response rates after multiple surveys, we adopted short measurements of the outcomes. The primary outcome only included two items for measuring FV intake, which were comparable with the measurements used in population-based studies in Hong Kong. The validity of the measurement should be established in future studies by testing its correlations with other instruments of fruit and vegetable intake. Thirdly, the follow-up period of 6 weeks was relatively short. Booster interventions are probably needed for sustained effects. Fourth, our sample was mostly women because many more mothers than fathers used family services and attended school programmes. Therefore, the effectiveness of the intervention on fathers warrants further study. Given the difficulties in recruiting fathers, and since mothers may have a stronger influence on the family diet, targeting mothers seems to be an effective strategy. Fifth, although we included only one intervention session, the level of engagement was not documented. However, in general, the participants were engaged with the interventions as reflected by the qualitative feedback. Lastly, our sample had a higher FV intake than the general public, probably because family service users and parents who are active in school programmes might be more health conscious. As the trial included MALC interventions, we did not select or exclude subjects by FV intake so that our results could be applicable to more parents. Five servings of FV are the recommended minimal intake, but more intake could have better health outcomes—and our intervention was effective in further increasing FV intake.

## 5. Conclusions

The present study used a cluster RCT design, adopted a population-based approach, and was among the first studies to use the HAPA model to develop single-session preventive interventions to effectively increase fruit and vegetable consumption in parents. Parallel changes in postulated variables that promote behavioural changes suggest that designs based on the HAPA framework may be useful in promoting other health-related behaviours. To broaden the effect at the population level, regular territory-wide interventions can be conducted to improve knowledge of the actual serving size, to promote FV intake, and to enhance food literacy. Training programmes for social workers are low-cost and should be scaled up to benefit more people.

## Figures and Tables

**Table 1:** Demographic characteristics across two arms.

Characteristics	Categories	MALC(*n* = 606)	FV(*n* = 197)	*p*
Sex, *n* (%)	Male	57 (9.4)	21 (10.7)	0.27
	Female	549 (90.6)	176 (89.3)	
Age, Mean (SD)		41.3 (5.9)	41.2 (6.3)	0.84
Marital status, *n* (%) ^1^	Never married	4 (0.7)	1 (0.5)	0.35
	Now married	553 (91.3)	176 (89.3)	
	Divorced/widowed/separated	45 (7.4)	17 (8.6)	
	Others	2 (0.3)	3 (1.5)	
Number of children, Mean (SD)		1.8 (0.7)	2.0 (0.9)	<0.001
Place of birth, *n* (%) ^2^	Hong Kong	248 (40.9)	69 (35.0)	0.36
	Guangdong province	184 (30.4)	73 (37.1)	
	Other provinces	166 (27.4)	51 (25.9)	
	Other countries	7 (1.2)	3 (1.5)	
Length of stay in Hong Kong, *n* (%) ^3^	≤1 year	33 (5.5)	13 (6.6)	0.50
	2–3 years	40 (6.6)	10 (5.1)	
	4–6 years	93 (15.4)	26 (13.2)	
	≥7 years	435 (71.8)	144 (73.1)	
Education, *n* (%) ^4^	Primary or below	55 (9.1)	22 (11.2)	0.02
	Secondary	416 (68.9)	149 (75.6)	
	Tertiary	133 (22.0)	26 (13.2)	
Family monthly income (HK$), *n* (%) ^5^	<10,000	117 (20.0)	55 (29.1)	<0.001
	10,000–19,999	204 (34.9)	79 (41.8)	
	≥20,000	264 (45.1)	55 (29.1)	

MALC: more appreciation or less criticism. FV: fruit and vegetable. ^1^ *n* (missing): MALC = 2. ^2^ *n* (missing): MALC = 1, FV = 1. ^3^ *n* (missing): MALC = 5, FV = 4. ^4^ *n* (missing): MALC = 2. ^5^ *n* (missing): MALC = 21, FV = 8. The table showing demographic characteristics across three arms has been published [24].

**Table 2:** Effects of the FV intervention at different time points (intention-to-treat analysis).

		Mean (SD)	FV vs. MALC		
	FV (*n* = 197)	MALC (*n* = 606)	BMD (95% CI)	ES	*p*
Fruit and vegetable intake per day in the past week, number of servings ^1^					
Baseline	5.41 (2.70)	4.09 (2.62)			
2-week follow-up	5.59 (1.92) *	3.64 (1.80) **	1.78 (1.44, 2.13)	0.97	<0.001
6-week follow-up	5.97 (2.22) **	3.77 (1.61) **	1.92 (1.43, 2.40)	1.08	<0.001
Fruit and vegetable intake per day in the past week, number of servings (with interaction term) ^2^					
2-week follow-up	5.59 (1.92) *	3.64 (1.80) **	1.51 (0.91, 2.11)	0.83	<0.001
6-week follow-up	5.97 (2.22) **	3.77 (1.61) **	1.81 (1.20, 2.42)	1.02	<0.001
Fruit and vegetable intake per day in the past week, number of servings (with HAPA variables)					
2-week follow-up ^3^	5.59 (1.92) *	3.64 (1.80) **	1.77 (1.42, 2.12)	0.97	<0.001
6-week follow-up ^4^	5.97 (2.22) **	3.77 (1.61) **	1.90 (1.39, 2.42)	1.07	<0.001
Fruit and vegetable intake per day in the past week, number of servings (per-protocol analysis)					
Baseline	5.54 (2.77)	3.99 (2.53)			
2-week follow-up	5.71 (2.00)	3.70 (1.86)	1.82 (1.39, 2.24)	0.96	<0.001
6-week follow-up	5.97 (2.39)	3.77 (1.52)	1.94 (1.57,2.30)	1.10	<0.001
Fruit intake per day in the past week, number of servings					
Baseline	2.29 (1.59)	1.81 (1.33)			
2-week follow-up	2.70 (1.28) **	1.69 (1.00) **	0.93 (0.68, 1.18)	0.86	<0.001
6-week follow-up	2.70 (1.20) **	1.79 (0.91)	0.79 (0.50, 1.08)	0.80	0.001
Vegetable intake per day in the past week, number of servings					
Baseline	3.13 (1.55)	2.28 (1.56)			
2-week follow-up	2.94 (1.13) **	1.97 (1.09) **	0.88 (0.66, 1.09)	0.80	<0.001
6-week follow-up	3.29 (1.43) **	1.97 (1.03) **	1.17 (0.89, 1.45)	1.03	<0.001
Outcome expectancies					
Baseline	9.02 (1.47)	9.14 (1.31)			
Immediate post-intervention	9.40 (1.02) **	9.19 (1.20) **	0.30 (0.15, 0.44)	0.26	<0.001
Intention					
Baseline	8.48 (1.73)	8.67 (1.58)			
Immediate post-intervention	9.17 (1.19) **	8.83 (1.66) **	0.50 (0.31, 0.69)	0.32	<0.001
2-week follow-up	8.37 (1.55)	7.56 (2.00) **	0.82 (0.49, 1.16)	0.43	<0.001
Self-efficacy					
Baseline	8.22 (1.85)	8.59 (1.67)			
Immediate post-intervention	8.99 (1.38) **	8.78 (1.54) **	0.46 (0.27, 0.65)	0.31	<0.001
2-week follow-up	8.08 (1.50) *	7.52 (1.93) **	0.64 (0.30, 0.99)	0.35	<0.001
Action planning					
Baseline	7.60 (2.02)	8.18 (1.63)			
Immediate post-intervention	8.69 (1.44) **	8.56 (1.90) **	0.44 (0.23, 0.65)	0.24	<0.001
2-week follow-up	7.97 (1.62) **	7.46 (1.92) **	0.69 (0.36, 1.02)	0.37	<0.001
6-week follow-up	8.07 (1.57) **	7.58 (1.81) **	0.61 (0.23, 0.99)	0.35	0.003
Coping planning					
Baseline	7.60 (2.16)	8.15 (1.94)			
Immediate post-intervention	8.60 (1.60) **	8.54 (1.64) **	0.37 (0.15, 0.59)	0.19	0.001
2-week follow-up	7.81 (1.72) *	7.35 (1.99) **	0.63 (0.30, 0.96)	0.32	<0.001
6-week follow-up	7.87 (1.72) **	7.52 (1.88) **	0.45 (0.13, 0.76)	0.28	0.007

FV: fruit and vegetable. MALC: more appreciation or less criticism. BMD: between-group mean difference. ES: effect size (Cohen’s *d*, small: 0.2–0.5; medium: 0.5–0.8; large: > 0.8). HAPA: health action process approach. * or ** marked below each arm: significant within-group differences compared with baseline. * *p* < 0.05; ** *p* < 0.01. ^1^ Adjusted for FV intake at baseline, educational level, income, and number of children. The arm, FV intake at baseline, educational level, and income had significant impact on the outcomes. ^2^ Adjusted for FV intake at baseline, educational level, income, and number of children and interaction term of arm and FV intake at baseline. The arm, FV intake at baseline, educational level, and income had significant impact on the outcomes. ^3^ Adjusted for FV intake at baseline, educational level, income, and number of children and HAPA variables (outcome expectancies, intention, self-efficacy, action planning and coping planning) measured immediately after the intervention. The arm, FV intake at baseline, educational level, and income had significant impact on the outcomes. ^4^ Adjusted for FV intake at baseline, educational level, income, and number of children and HAPA variables (outcome expectancies, intention, self-efficacy, action planning, and coping planning) measured immediately after the intervention and after 2 weeks (if available). The arm, FV intake at baseline, educational level, and income had significant impact on the outcomes.

## Data Availability

Data cannot be shared publicly due to the ethical restrictions. Applications for additional data sharing could be sent to the corresponding authors.

## Supplementary Materials

The following are available online at https://www.mdpi.com/article/10.3390/ijerph18105206/s1, Table S1: Effects of the FV intervention at different time points (intention-to-treat analysis in three groups), Table S2: Effects of the FV intervention at different time points (per-protocol analysis in three groups), Table S3: Effects of the FV intervention at different time points (per-protocol analysis in two groups).
