# Supplementary material for: Promoting Fruit and Vegetable Intake in Parents: A Cluster Randomised Controlled Trial
Source: Int J Environ Res Public Health. 2021 May 13;18(10):5206. doi: 10.3390/ijerph18105206 (PMC8153553; doi:10.3390/ijerph18105206)
Supplement: Supplementary file 1 [file ijerph-18-05206-s001.zip › Supplementary File_20210513.pdf]

Table S1. Effects of the FV intervention at different time points (intention-to-treat analysis in three groups)

	Mean (SD)			FV vs MA		FV vs LC	
	FV (n = 197)	MA (n = 270)	LC (n = 336)	BMD (95% CI)	ES	BMD (95% CI)	ES
Fruit and vegetable intake per day in the past week, number of servings							
T1	5.41 (2.70)	4.21 (2.82)	4.00 (2.46)				
T3	5.63 (1.94)*	3.50 (1.55)**	3.82 (1.98)**	1.97 (1.48, 2.46)**	1.14	1.65 (1.16, 2.14)**	0.84
T4	5.92 (2.23)**	3.67 (1.57)**	3.88 (1.66)	2.02 (1.40, 2.64)**	1.08	1.80 (1.18, 2.43)**	0.95
Outcome expectancies							
T1	9.02 (1.47)	9.25 (1.23)	9.06 (1.36)				
T2	9.40 (1.02)**	9.34 (1.06)**	9.07 (1.28)	0.21 (0.05, 0.37)*	0.20	0.36 (0.21, 0.52)**	0.30
Intention							
T1	8.48 (1.73)	8.82 (1.57)	8.55 (1.72)				
T2	9.17 (1.19)**	8.95 (1.52)**	8.73 (1.62)**	0.47 (0.25, 0.69)**	0.34	0.53 (0.32, 0.73)**	0.36
T3	8.40 (1.55)	7.67 (2.10)**	7.54 (1.91)**	0.75 (0.33, 1.17)**	0.40	0.82 (0.43, 1.21)**	0.43
Self-efficacy							
T1	8.22 (1.85)	8.73 (1.60)	8.47 (1.71)				
T2	8.99 (1.38)**	8.94 (1.40)**	8.64 (1.64)**	0.38 (0.15, 0.60)**	0.20	0.52 (0.31, 0.73)**	0.34
T3	8.12 (1.53)	7.60 (2.04)**	7.51 (1.85)**	0.63 (0.24, 1.01)**	0.34	0.66 (0.29, 1.02)**	0.38
Action planning							
T1	7.60 (2.02)	8.30 (1.92)	8.09 (1.87)				
T2	8.69 (1.44)**	8.71 (1.51)**	8.45 (1.71)**	0.35 (0.11, 0.59)**	0.24	0.51 (0.28, 0.73)**	0.32
T3	7.97 (1.68)**	7.59 (1.98)**	7.40 (1.90)**	0.57 (0.15, 0.98)**	0.31	0.71 (0.31, 1.11)**	0.39
T4	8.13 (1.58)**	7.65 (1.85)**	7.55 (1.82)**	0.64 (0.17, 1.12)*	0.37	0.70 (0.33, 1.07)**	0.40
Coping planning							
T1	7.60 (2.16)	8.25 (1.96)	8.06 (1.91)				
T2	8.60 (1.60)**	8.66 (1.57)**	8.44 (1.69)**	0.31 (0.06, 0.56)*	0.19	0.42 (0.06, 0.56)**	0.25
T3	7.84 (1.76)*	7.39 (2.12)**	7.34 (1.91)**	0.61 (0.16, 1.06)**	0.31	0.63 (0.21, 1.05)**	0.34
T4	7.90 (1.70)**	7.50 (1.98)**	7.58 (1.81)**	0.51 (0.03, 0.99)*	0.27	0.40 (-0.01, 0.82)	0.23

MA: more appreciation. LC: less criticism. FV: fruit and vegetable. BMD: between-group mean difference. ES: effect size. T1: baseline. T2: immediate post-intervention. T3: 2-week follow-up. T4: 6-week follow-up. *$p < 0.05$; ** $p < 0.01$. * or **marked below each arm: significant within-group differences compared with T1.

Table S2. Effects of the FV intervention at different time points (per-protocol analysis in three groups)

	Mean (SD)			FV vs MA		FV vs LC	
	FV (n = 120)	MA (n = 169)	LC (n = 225)	BMD (95% CI)	ES	BMD (95% CI)	ES
Fruit and vegetable intake per day in the past week, number of servings							
T1	5.54 (2.77)	4.08 (2.71)	3.92 (2.39)				
T3	5.71 (2.00)	3.52 (1.46)**	3.84 (2.10)	1.99 (1.52, 2.46)**	1.17	1.67 (1.23, 2.12)**	0.81
T4	5.97 (2.39)	3.66 (1.46)	3.84 (1.56)	2.04 (1.63, 2.45)**	1.07	1.86 (1.47, 2.25)**	0.98
Outcome expectancies							
T1	9.07 (1.42)	9.32 (1.13)	9.02 (1.30)				
T2	9.43 (1.08)**	9.32 (1.10)	9.04 (1.30)	0.30 (0.10, 0.50)**	0.27	0.40 (0.21, 0.58)**	0.33
Intention							
T1	8.57 (1.59)	8.93 (1.44)	8.49 (1.65)				
T2	9.22 (1.20)**	8.95 (1.53)	8.69 (1.65)*	0.57 (0.29, 0.85)**	0.41	0.54 (0.28, 0.80)**	0.36
T3	8.27 (1.47)	7.56 (2.13)**	7.52 (1.93)**	0.80 (0.32, 1.28)**	0.42	0.70 (0.26, 1.15)**	0.39
Self-efficacy							
T1	8.28 (1.79)	8.81 (1.52)	8.44 (1.65)				
T2	9.12 (1.24)**	9.00 (1.41)*	8.61 (1.64)*	0.51 (0.25, 0.78)**	0.38	0.66 (0.42, 0.91)**	0.44
T3	8.13 (1.44)	7.51 (2.09)**	7.44 (1.88)**	0.79 (0.34, 1.25)**	0.43	0.75 (0.32, 1.17)**	0.43
Action planning							
T1	7.56 (2.10)	8.47 (1.81)	8.03 (1.88)				
T2	8.77 (1.45)**	8.75 (1.52)**	8.44 (1.67)**	0.54 (0.23, 0.84)**	0.36	0.62 (0.34, 0.90)**	0.39
T3	8.00 (1.53)*	7.47 (1.98)**	7.31 (1.92)**	0.83 (0.42, 1.25)**	0.46	0.86 (0.47, 1.25)**	0.48
T4	8.11 (1.46)**	7.67 (1.85)**	7.51 (1.82)**	0.73 (0.31, 1.14)**	0.43	0.75 (0.37, 1.14)**	0.44
Coping planning							
T1	7.61 (1.59)	8.41 (1.88)	8.04 (1.89)				
T2	8.73 (1.59)**	8.72 (1.60)**	8.44 (1.66)**	0.50 (0.17, 0.83)**	0.31	0.58 (0.27, 0.89)**	0.35
T3	7.85 (1.69)	7.25 (2.17)**	7.26 (1.94)**	0.85 (0.36, 1.33)**	0.43	0.73 (0.28, 1.19)**	0.39
T4	7.90 (1.64)	7.52 (2.02)**	7.53 (1.79)**	0.61 (0.15, 1.06)**	0.33	0.50 (0.08, 0.93)*	0.29

MA: more appreciation. LC: less criticism. FV: fruit and vegetable. BMD: between-group mean difference. ES: effect size. T1: baseline. T2: immediate post-intervention. T3: 2-week follow-up. T4: 6-week follow-up. *$p < 0.05$; ** $p < 0.01$. * or **marked below each arm: significant within-group differences compared with T1.

Table S3. Effects of the FV intervention at different time points (per-protocol analysis in two groups)

	Mean (SD)		FV vs MALC		
	FV (n = 120)	MALC (n = 394)	BMD (95% CI)	ES	*p*
Outcome expectancies					
T1	9.07 (1.42)	9.15 (1.26)			
T2	9.43 (1.08)**	9.16 (1.23)	0.36 (0.18, 0.53)	0.30	<0.001
Intention					
T1	8.57 (1.59)	8.68 (1.58)			
T2	9.22 (1.20)**	8.80 (1.60)	0.55 (0.31, 0.80)	0.36	<0.001
T3	8.27 (1.47)	7.53 (2.03)**	0.74 (0.33, 1.16)	0.39	<0.001
Self-efficacy					
T1	8.28 (1.79)	8.60 (1.60)			
T2	9.12 (1.24)**	8.78 (1.56)**	0.60 (0.40, 0.83)	0.40	<0.001
T3	8.13 (1.44)	7.47 (1.97)**	0.77 (0.37, 1.16)	0.41	<0.001
Action planning					
T1	7.56 (2.10)	8.22 (1.61)			
T2	8.77 (1.45)**	8.57 (1.86)**	0.58 (0.32, 0.85)	0.33	<0.001
T3	8.00 (1.53)*	7.38 (1.94)**	0.85 (0.48, 1.21)	0.46	<0.001
T4	8.11 (1.46)**	7.58 (1.83)**	0.74 (0.38, 1.10)	0.42	<0.001
Coping planning					
T1	7.60 (2.15)	8.20 (1.89)			
T2	8.73 (1.59)**	8.56 (1.64)**	0.55 (0.26, 0.83)	0.34	<0.001
T3	7.85 (1.69)	7.26 (2.03)**	0.78 (0.36, 1.20)	0.40	<0.001
T4	7.90 (1.64)	7.53 (1.89)**	0.55 (0.14, 0.84)	0.30	0.007

FV: fruit and vegetable. MALC: more appreciation or less criticism. BMD: between-group mean difference. ES: effect size. T1: baseline. T2: immediate post-intervention. T3: 2-week follow-up. T4: 6-week follow-up. *$p < 0.05$; ** $p < 0.01$. * or **marked below each arm: significant within-group differences compared with T1.
